# Mitochondrial Acetyl‐CoA Synthetase 3 is Biosignature of Gastric Cancer Progression

**DOI:** 10.1002/cam4.1295

**Published:** 2018-03-01

**Authors:** Wei‐Chun Chang, Wei‐Chung Cheng, Bi‐Hua Cheng, Lumin Chen, Li‐Jing Ju, Yu‐Jer Ou, Long‐Bin Jeng, Mei‐Due Yang, Yao‐Ching Hung, Wen‐Lung Ma

**Affiliations:** ^1^ Sex Hormone Research Center Department of Obstetrics and Gynecology Research Center for Tumor Medical Science Taichung 40403 Taiwan; ^2^ Department of Gastroenterology China Medical University/Hospital China Medical University Taichung 40403 Taiwan; ^3^ Department of Surgery China Medical University/Hospital China Medical University Taichung Taichung 40403 Taiwan; ^4^ Graduate Institution of Clinical Medical Science, Graduate Institute of Biomedical Sciences, and Graduate Institution of Cancer Biology, School of Medicine China Medical University Taichung 40403 Taiwan; ^5^ Department of OBs & GYN Chia‐Yi Chang‐Gung Memorial Hospital Chang Gung University College of Medicine Chia‐Yi Taiwan; ^6^ Department of OBs & GYN, BenQ Medical Center Nanjing Medical University Suzhou Jiangsu Province 215004 China; ^7^ Department of OBs & GYN Kaohsiung Chang‐Gung Memorial Hospital Kaohsiung Taiwan; ^8^ Department of Nursing Asia University Taichung 41354 Taiwan

**Keywords:** acyl‐coA synthetase superfamily 3, Cholesterol, Gastric Cancer, Mevalonate pathway

## Abstract

Cholesterol affects cancer progression, and acetyl‐CoA is the primary cholesterogenesis substrate. The previous work has defined cholesterol bioflux via lipoprotein/receptor route is the gastric cancer (GCa) prognosis biosignature. The prognosis importance of acetyl‐CoA to cholesterogenesis (mevalonate pathway) in GCa is yet to be defined. Using Kaplan–Meier Plotter web‐based gene survival analyzer and The Cancer Genome Atlas (TCGA)‐database analyzed with DBdriver.v2 platform, we revealed acetyl‐CoA production and the mevalonate pathway are associated with GCa prognosis. We found mitochondrial‐derived acetyl‐CoA contributing enzymes (acyl‐coA synthetase super‐family 3; ACSS3) is the GCa progression confounder. Interestingly, it is not HMGCR (the committee enzyme of mevalonate pathway), but lower mevalonate pathway enzymes (e.g., MVK, LSS, DHCR14A1, SC4MOL, HSD17B7, SC5D) promote GCa patients 5‐years overall survival in a differential level. Advanced analyses found ACSS3 is prognosis biosignatures for multiple GCa disease conditions. This report uncovered a higher expression of ACSS3 in tumor comparing to normal parental lesions, which implicates a targeting value for GCa therapy. While knockdown ACSS3 could suppress growth and invasion of GCa cells, of which even more impactful under starvation condition. This is the first report, surprisingly, revealed ACSS3 as important cancer prognosis biomarker. Targeting ACSS3 could be a novel therapeutic strategy for cancer, in this case, GCa.

## Introduction

Gastric cancer (GCa) is the third leading cause of cancer death worldwide (World Health Organization, Cancer: Fact Sheet No 297; http://www.who.int/mediacentre/factsheets/fs297/en/). The high mortality rates of GCa could be due to late diagnosis 1 and few effective adjuvant therapy agents available for patients 2. The prognosis is poor for advanced GCa patients receiving gastrectomy, and the 5‐year recurrence‐free survival rate is only around 25% 3. In addition, few chemotherapy options are available for postsurgery patients 4, 5. The therapeutic efficacy of surgery combined with chemotherapy in GCa patients is limited according to a meta‐analysis 6. Therefore, there is clearly an unmet medical need in GCa patient management.

Cholesterol is a major component of cell membranes and a vital metabolic material for fast‐growing cancer cells. A previous study has shown the importance of low‐density lipoprotein (LDL) and its cholesterol‐shuttling receptor (LDLR; L/R route), which facilitate steroidogenesis and sex hormone receptor action, and promote GCa progression 7. The study indicated that the L/R route is a GCa progression biomarker. However, the importance of endogenous de novo cholesterogenesis in GCa prognosis is unknown.

The mevalonate pathway is an enzymatic cascade responsible for de novo cholesterogenesis. The anabolism starts with the supply of acetyl‐CoA, which may have two possible resources. The first resource is the acetyl‐CoA matrix (nonmitochondria), which is catalyzed by ACSS2 (acetyl‐CoA synthetase 2; acetate from extracellular pool), PDHB (pyruvate dehydrogenase; intermediate step of glycolysis), and ACAT1/2 (thiolase; final step of fatty acid *β*‐oxidation). The second acetyl‐CoA resource is derived from mitochondrial ACSS1/3 (acetyl‐CoA synthetase 1/3) and ACLY (ATP citrate lyase).

The first enzymatic action for cholesterogenesis is condensing acetyl‐CoA and acetoacetyl‐CoA by thiolation (HMGCS; hydroxymethylglutaryl‐coA synthase) to generate 3‐hydroxy‐3‐methylglutaryl‐CoA (HMG‐CoA). The next step is thiolation to convert 3‐hydroxy‐3‐methylglutaryl‐CoA to mevalonate by HMGCR (HMG‐CoA reductase), which is recognized as a rate‐limiting step of cholesterogenesis. There are then 12 more steps to produce cholesterol. The intermediates of those enzymatic steps are reported to be involved in various biological actions, including inflammation, cell migration, and differentiation. Because of the enzymatic cascade of cholesterogenesis from various sources of acetyl‐CoA and the following mevalonate pathway, identifying the resources of acetyl‐CoA and the roles of mevalonate pathway enzymes that determine GCa progression might provide a potential target against cancer cells.

Web‐based gene survival analysis was performed to analyze the enzymes that produce acetyl‐CoA and the enzymes corresponding to the mevalonate pathway in patients. The strategy involves meta‐analysis of online cDNA microarray databases that predict the outcome in appropriately powered cohorts and provide a feasible, unbiased, and genome‐wide approach to analyze genes in cancer progression 8, 9,http://kmplot.com/analysis/index.php?p=service&cancer=gastric). We used a web‐based survival analyzer (Kaplan–Meier plotter) to test candidate genes in GCa disease survival and calculate the importance of gene clusters in GCa patients with unmet medical needs.

## Methods

### Meta‐analysis of gastric cancer patient with Kaplan–Meier plotter OS analyzer

We analyzed the 5‐year OS rates of GCa using the web‐based gene survival analyzer Kaplan–Meier plotter (http://kmplot.com/analysis/index.php?p=service&cancer=gastric) 9. The 5‐year OS was assessed in all GCa cohorts stratified by median classifier expression. GCa subtypes included all patients (nonclassified; *n* = 876) and surgery (*n* = 380), surgery and 5′FU treatment (5FU and surgery; *n* = 153), HER2– (*n* = 532), and HER2 +  (*n* = 344) patients. The input genes and classifiers are as follows: acetyl‐CoA synthesis enzymes: 211023_at (PDHB), 201128_s_at (ACLY), 219616_at (ACSS3), 218322_s_at (ACSS2), 201661_s_at (FACL3), 205412_at (ACAT1), 209608_s_at (ACAT2); cholesterogenesis enzymes: 221750_at (HMGCS1), 202540_s_at (HMGCR), 36907_at (MVK), 203515_s_at (PMK), 203027_s_at (MVD), 201275_at (FDPS), 208647_at (FDFT1), 202245_at (LSS), 215093_at (NSDHL), 201791_s_at (DHCR7), 202314_at (P450‐14DM), 210130_s_at (TM7SF2), 201795_at (LBR), 209146_at (SC4MOL), 220081_x_at (HSD17B7), 202735_at (EBP), 215064_at (SC5DL), 200862_at (DHCR24),

### Scoring method of hazard ratio (HR) summation to evaluate targeting value (HR score)

In previous work, we developed a formula to calculate the impact of genes on different GCa conditions with the KM plotter survival analyzer 7:

$$\begin{array}{cc} \text{HR score} & =(\text{Avg. of HR of gene sets})\\ & =\frac{\sum ({\text{HR}}_{n-1})\times (-{\text{log}}_{10}(\text{p}-\text{value}))}{\text{n}} \end{array}$$

In order to evaluate the impact of each genes, the absolute value of HR minus 1. For adjusting the effect of genes, it is multiplied by negative log_10_(*P*‐value) to balance their importance. The summed score is divided by the number of genes and multiplied by 100 to obtain the HR score, or the average HR of genes. We set the threshold as 300 to indicate the significance of genes. An HR score> 300 could be considered as significant for targeting, whereas an HR score ≤300 indicates less value for targeting in GCa therapy.

### Meta‐analysis of gastric cancer patients with TCGA database

Previous developed DriverDB (http://ngs.ym.edu.tw/driverdb), a database that incorporates more than 9500 cancer‐related RNA‐seq datasets and more than 7000 exome‐seq. datasets from TCGA, the International Cancer Genome Consortium (ICGC), and published papers 10, 11, were used in this study. In DriverDB, there are 420 primary tumors and 37 adjacent normal tissues (including 34 normal‐tumor pairs) in the gastric cancer dataset of TCGA. We validated the expression of indicated genes in nontumor (NT) versus tumor parental (TP) tissues in a paired or nonpaired fashion. We used a student's t‐test to compare the mean expression levels of genes between primary tumors and adjacent normal tissues, and we used a paired *t*‐test for matched normal and tumor pair samples. A *P*‐value less than 0.05 was considered statistically significant.

### Cell maintenance, ACSS3 knockdown by lentivirus‐based gene silencing, and reagents

SNU1, AGS GCa, and HEK293T cells were purchased from the Food Industry Research and Development Institute in Taiwan (BCRC purchase number: 60210). The cells were maintained in DMEM with 10% FCS (Invitrogen), 1% L‐glutamine, and 1% penicillin/streptomycin, as described previously 12.

#### Lentiviral‐based gene delivery

Gene silencing using shRNA toward ACSS3 (shACSS3) and luciferase (shLuc) shRNA was used as reported previously 13. The pLKO‐shLuciferase and shACSS3 (TRCN0000152146) plasmids were obtained from the National RNAi Core Facility Platform (Institute of Molecular Biology/Genome Research Center, Academia Sinica, supported by the National Core Facility Program for Biotechnology; grant MOST104‐2319‐B‐001‐001). The lentiviral production and infection procedures used in this study followed a previous report 14. In brief, psPAX2 (packaging plasmid) and pMD2G (envelope plasmid) (Addgene) were cotransfected into HEK293T cells. We then harvested virus‐containing media to infect the GCa cells. After 48 h of infection, we used puromycin (5 *μ*g/mL) to select a positive infection. After selection for 2‐weeks, gene expression and biological assays were performed.

#### Apoptosis assay

The apoptosis assay was conducted as reported previously 15. Briefly, 10^6^ cells were cultured in a 100‐mm dish with or without 10 nmol/L DHT for 24 h. They were then trypsinized, washed, and stained with fresh 5 *μ*mol/L propidium iodine (PI) in PBS. The dead cells were then detected by flowcytometry (BD, SLRII) and analyzed by FlowJo 7.6 software.

### Colony‐forming assay

Colony‐forming assays were performed as previously reported 16. Briefly, 1 × 10^4^ cells/dish were seeded onto 3.5‐cm plates with DMEM in 10% FBS with various treatments for 7 days. After treatments, 1/3 of the total volume of the 10% formaldehyde solution was added to fix the cells, which were then allowed to stain with Crystal Violet for 5 min**.** After washing with PBA, the colonies were photographed.

#### Wound‐healing cell migration assay

The procedure of measuring cell migration ability was wound‐healing assays, which modified from previous publication 17. In brief, cell seed on plate till around 80% confluence, a 200‐*μ*L pipette tip was then used to create a linear wound area. Photographs were taken under a light microscope at 0‐h. We observed wound closure for 24‐h and then took photographs. Migration activity was defined by subtracting the wound area (*μ*m^2^) at 0‐h from that at 24‐h. The photographic images were analyzed by NIS Elements BR3.1v software (Nikon).

#### Statistics

In addition to the Kaplan–Meier plotter website calculation, a paired *t*‐test was used for biological assays, and the standard error of mean (SEM) served as an experimental variation. *P*‐values less than 0.05 were considered to be statistically significant.

## Result

### Mitochondrial ACSS3 contributes to acetyl‐CoA matrix and promotes GCa progression

**Figure 1:**
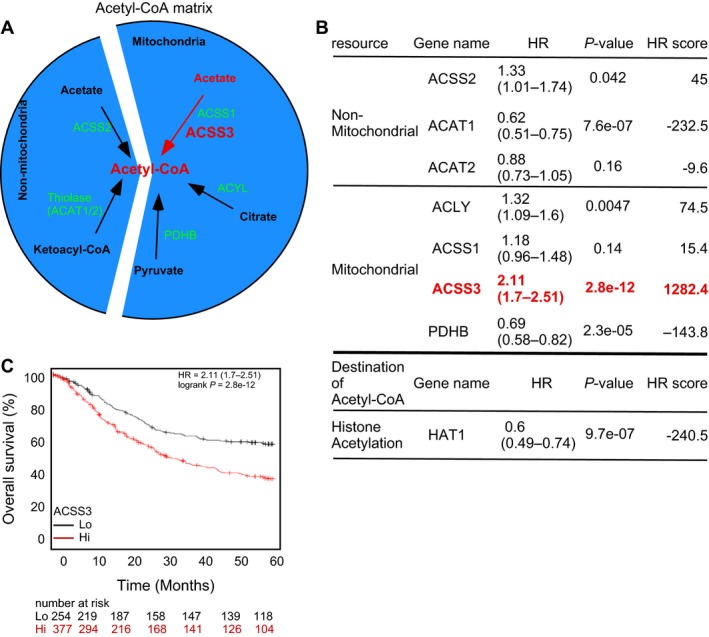
The mitochondrial acetyl‐CoA contributor ACSS3 is a GCa progression gatekeeper. (**A)**. Illustration of acetyl‐CoA matrix in a cell. The cellular acetyl‐CoA is contributed by mitochondrial and nonmitochondrial sources. The mitochondrial source is a major resource of acetyl‐CoA, which is converted from either acetate, citrate, or pyruvate by ACSS1/3, ACYL, or PDHB, respectively. On the other hand, the nonmitochondrial‐derived acetyl‐CoA is converted from acetate or ketoacyl‐CoA by ACSS2 or ACAT1/2, respectively. (**B)**. Calculation of the HR score of acetyl‐CoA contributing enzymes in GCa progression. ACSS3 is considered to have the most impact as a GCa progression confounder, and the HR score threshold is set as >300. (**C)**. ACSS3 expression is associated with the 5‐year overall survival (OS; %) of GCa. The HR is 2.11 (range 1.7–2.51), the *P*‐value is 2.8e‐12, and the HR score is 1281.4.

To analyze the resources of acetyl‐CoA in GCa progression, we first illustrated the acetyl‐CoA production map in a cell. As shown in Figure 1A, there are two major resources (nonmitochondrial and mitochondrial) that contribute to the intracellular pool of acetyl‐CoA. When comparing nonmitochondrial‐derived enzymes with respect to GCa 5‐year overall survival (OS) using the KM plotter analyzer, we found that ACSS2, ACAT1, and ACAT2 (HR score = 45, −232.5, and −9.6, respectively) are irrelevant to GCa progression (Fig. 1B). When analyzing mitochondrial enzymes, we also found that ACLY, ACSS1, and PDHB (HR score = 74.1, 15.4, and −143.8, respectively) are also irrelevant to GCa progression. However, surprisingly, ACSS3 profoundly impacted GCa disease progression (HR = 2.11 (1.7–2.61); *P*‐value = 2.8e‐12; HR score = 1282.4; Fig. 1B and C). This result indicates that ACSS3 is the major acetyl‐CoA producer in GCa progression.

The metabolic destination of acetyl‐CoA could either contribute to acetylation resources for histone lysine residue or enter the mevalonate pathway. Thus, we analyzed the contribution of histone acetyl‐transferase (HAT1) in GCa 5‐year OS. The result shows that HAT1 is a negative factor for GCa progression (HR score = −240.5), indicating that the destination of ACSS3‐acetyl‐CoA is less likely to contribute to histone acetylation.

### ACSS3 and the lower‐mevalonate pathway are GCa progression biosignatures

**Figure 2:**
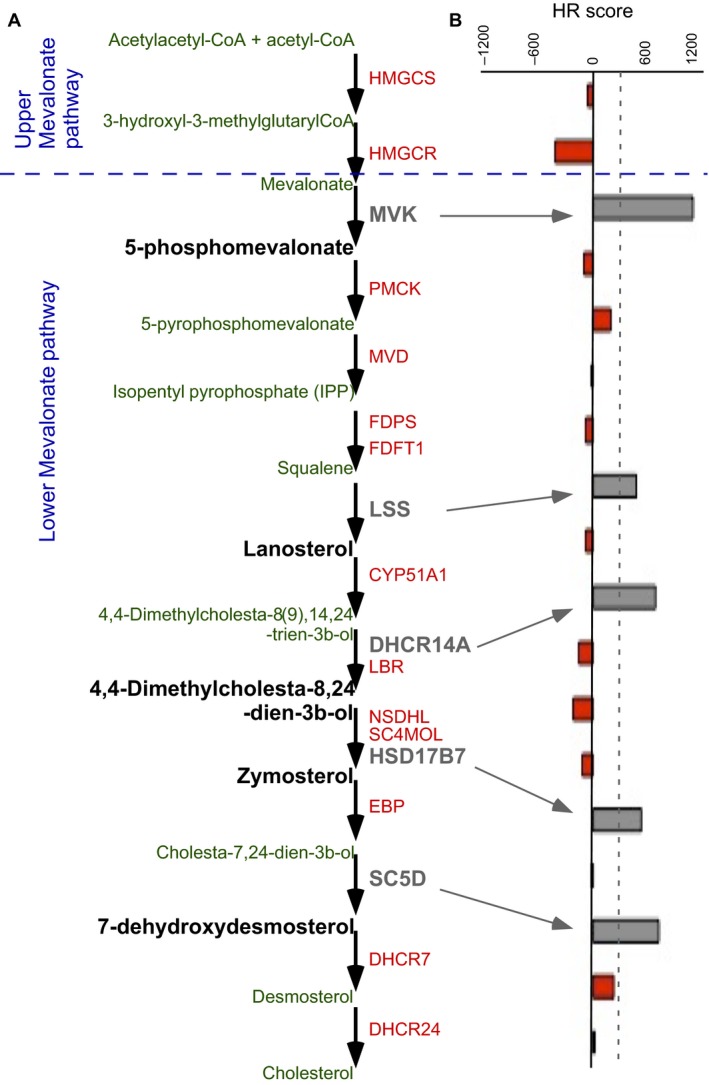
The lower‐mevalonate pathway enzymes are gatekeepers of GCa progression (**A**). Schematic illustration of mevalonate pathways, including the upper mevalonate pathway (HMGCS and HMGCR convert acetyl‐CoA to 3‐hydroxyl‐3‐3‐methylglutaryl‐CoA and mevalonate, respectively) and the lower mevalonate pathway (a series of enzymes that catalyze mevalonate to cholesterol). The enzymes (gray‐colored) significantly affect GCa progression (HR score > 300), and lipidomes are shown in black. The rest of the enzymes considered were not GCa progression gatekeepers and are shown in red, of which lipidomes are shown in green. (**B**). Bar‐graph of HR scores showing the associations of mevalonate‐enzymes in all GCa patients. The arrow points from significant enzymes in scheme A to the corresponding HR score in **B**. The dashed‐line indicates the threshold value (HR score >300) used to determine the significance of genes. Five enzymes (MVK, LSS, DHCR14A, HSD17B7, and SC5D) are considered as confounders of GCa progression.

Since acetyl‐CoA might go through the mevalonate pathway and influence GCa progression, the mevalonate pathway enzymes were compared with respect to GCa 5‐year OS. As shown in Figure 2A, the mevalonate pathway can be divided into upper‐ and lower‐mevalonate pathways. The upper‐mevalonate pathway is recognized as a rate‐limiting step that converts acetyl‐coA to mevalonate through HMGCS and HMGCR, which is a pharmacological target site of statins (cholesterol‐lowering drugs). Unexpectedly, the result showed that HMGCS and HMGCR are negatively associated with GCa progression (Fig. 2B; HR score = −60.8 and −356.5, respectively). We then analyzed lower‐mevalonate pathway enzymes and found that MVK, LSS, DHCR14A, HSD17B7, and SC5D are significantly positive factors (HR = 942.6, 407.3, 590.2, 455.9, and 619.9, respectively) that contribute to GCa progression.

**Figure 3:**
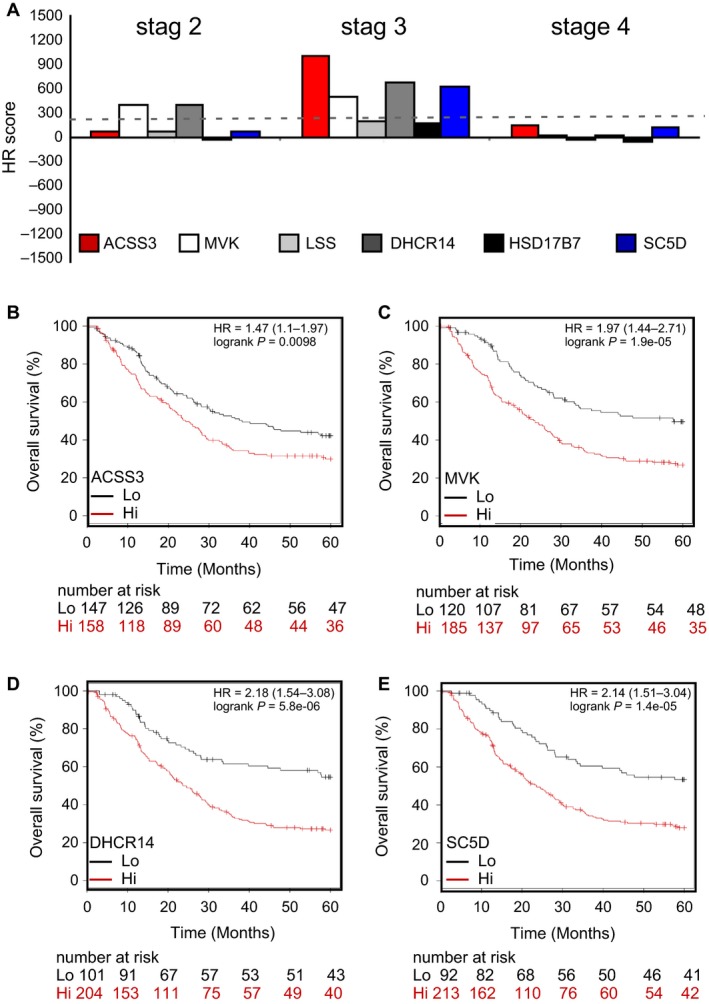
ACSS3, MVK, DHCR14, and SC5D are gatekeepers of stage 3 GCa patient progression. (**A)**. Association of the five enzymes ACSS3, MVK, LSS, DHCR14, HSD17B7, and SC5D in various stages of GCa patients. Bar‐graph of the HR score showing the associations of the five enzymesin GCa patients. Among these enzymes, ACSS3, MVK, DHCR14, and SC5D are considered to have the most impact on stage 3 GCa patients. (**B)**. ACSS3 expression is associated with stage 3 GCa 5‐year OS (%). The HR is 1.47 (range 1.1–1.97), and the *P*‐value is 0.0098. (**C)**. MVK expression is associated with stage 3 GCa 5‐year OS (%). The HR is 1.97 (range 1.44–2.71), and the *P*‐value is 1.9E‐05. (**D)**. DHCR14 expression is associated with stage 3 GCa 5‐year OS (%). The HR is 2.18 (range 1.54–3.08), and the *P*‐value is 5.8E‐06. **E**. SC5D expression is associated with stage 3 GCa 5‐year OS (%). The HR is 2.14 (range 1.51–3.04), and the *P*‐value is 1.4E‐05.

The survival benefit of surgery in GCa is best before stage 3 GCa develops 18. To evaluate the impact of genes on stages of GCa, we analyzed the ACSS3, MVK, LSS, DHCR14, HSD17B7, and SC5D genes and their association with various stages of GCa (Fig. 3A). We found that ACSS3 (Fig. 3B; HR score = 905.2), MVK (Fig. 3C; HR score = 458), DHCR14 (Fig. 3D; HR score = 617.9), and SC5D (Fig. 3E; HR score = 553.3) mostly impact stage 3 patient survival.

**Figure 4:**
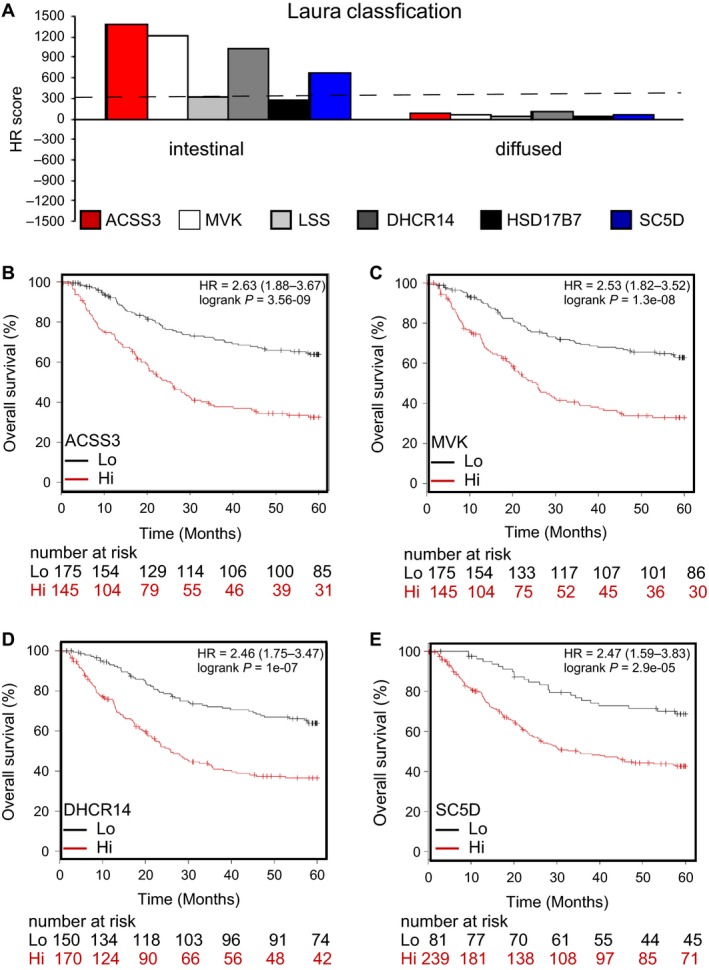
ACSS3, MVK, DHCR14, and SC5D are gatekeepers of intestinal‐type GCa progression. (**A)**. Association of the five enzymes ACSS3, MVK, LSS, DHCR14, HSD17B7, and SC5D in two histological types of GCa patients (intestinal and diffused types). HR score bar‐graph showing the associations of the five enzymes in GCa patients. Among these enzymes, ACSS3, MVK, DHCR14, and SC5D are considered to have the most impact on intestinal‐type GCa patients. (**B)**. ACSS3 expression is associated with intestinal‐type GCa 5‐year OS (%). The HR is 2.63 (range 1.88–3.67), and the *P*‐value is 3.56E‐09. (**C)**. MVK expression is associated with intestinal‐type GCa 5‐year OS (%). The HR is 2.53 (range 1.82–3.52), and the *P*‐value is 1.3E‐08. (**D)**. DHCR14 expression is associated with intestinal‐type GCa 5‐year OS (%). The HR is 2.46 (range 1.75–3.47), and the *P*‐value is 1E‐07. **(E)**. SC5D expression is associated with intestinal‐type GCa 5‐year OS (%). The HR is 2.47 (range 1.59–3.83), and the *P*‐value is 2.9E‐05.

Histological patterns are also a determinant of therapy outcome in GCa patient management. We analyzed the five genes in relation to the 5‐year OS of intestinal‐ (less malignant) and diffused‐type GCa (more malignant). We found that all five genes have less impact on the diffused type but on the intestinal type (Fig. 4A). The HR scores for ACSS3, MVK, DHCR14, and SC5D are 1376.3, 1206.6, 1022, and 667, respectively (Fig. 4B–E). These data suggest that lower‐mevalonate enzymes and ACSS3 might have therapeutic efficacy as indicators of GCa.

**Figure 5:**
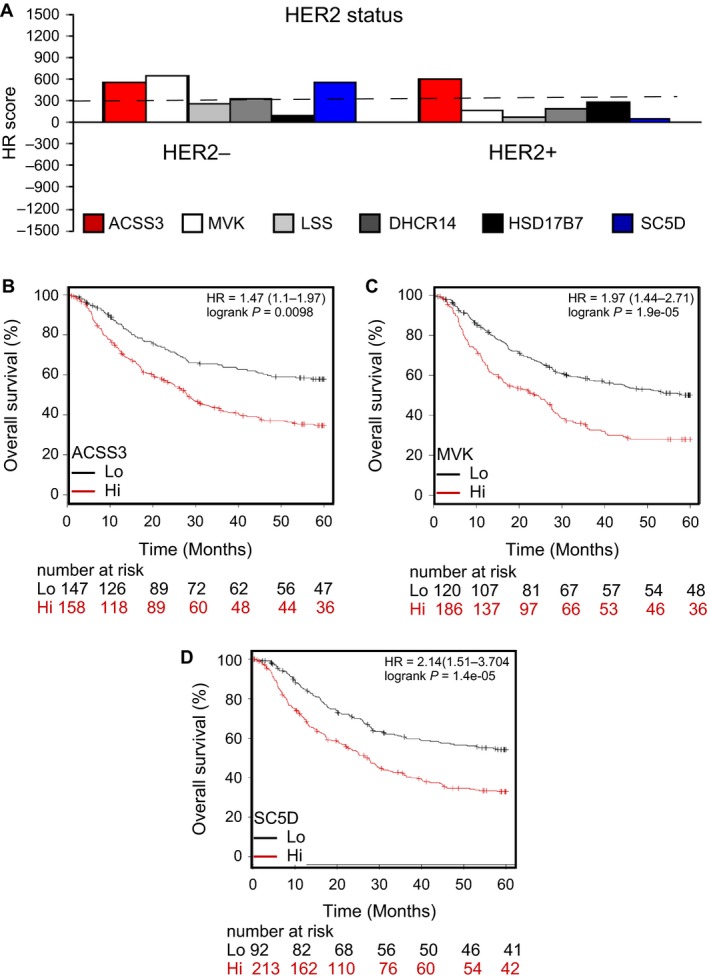
ACSS3, MVK, and SC5D are gatekeepers of GCa progression in HER2 negative (HER2–) patients. (**A)**. Association of the five enzymes ACSS3, MVK, LSS, DHCR14, HSD17B7, and SC5D in HER2 + /–GCa patients. Bar‐graph of HR score showing the associations of the five enzymes in GCa patients. Among these enzymes, ACSS3, MVK, and SC5D are considered to have the most impact on HER2– GCa patients. (**B)**. ACSS3 expression is associated with HER2– GCa 5‐year OS (%). The HR is 1.47 (range 1.1–1.97), and the *P*‐value is 0.0098. (**C)**. MVK expression is associated with HER2– GCa 5‐year OS (%). The HR is 1.97 (range 1.44–2.71), and the *P*‐value is 1.9E‐05. (**D)**. SC5D expression is associated with HER2– GCa 5‐year OS (%). The HR is 2.14 (range 1.51–3.74), and the *P*‐value is 1.4E‐05.

Other than surgery, nonresectable patients might receive anti‐HER2 therapy (e.g., trastuzumab) if the biopsy data show positive HER2 expression. However, there is little hope for nonresectable patients and those with negative HER2 expression. Therefore, the five genes were also analyzed for their association with the HER2 expression status of GCa patients. The result showed that only ACSS3 is a progression promoter for HER2 +  patients (HR score = 603.3) (Fig. 5A). ACSS3, MVK, and SC5D are HER2– progression promoters (HR score = 552, 652.7, and 549.8, respectively) (Fig. 5B–D). These data suggest that targeting ACSS3, MVK, and SC5D might be beneficial to HER2– GCa patients.

### Targeting ACSS3 for GCa therapy of unmet medical needs

**Figure 6:**
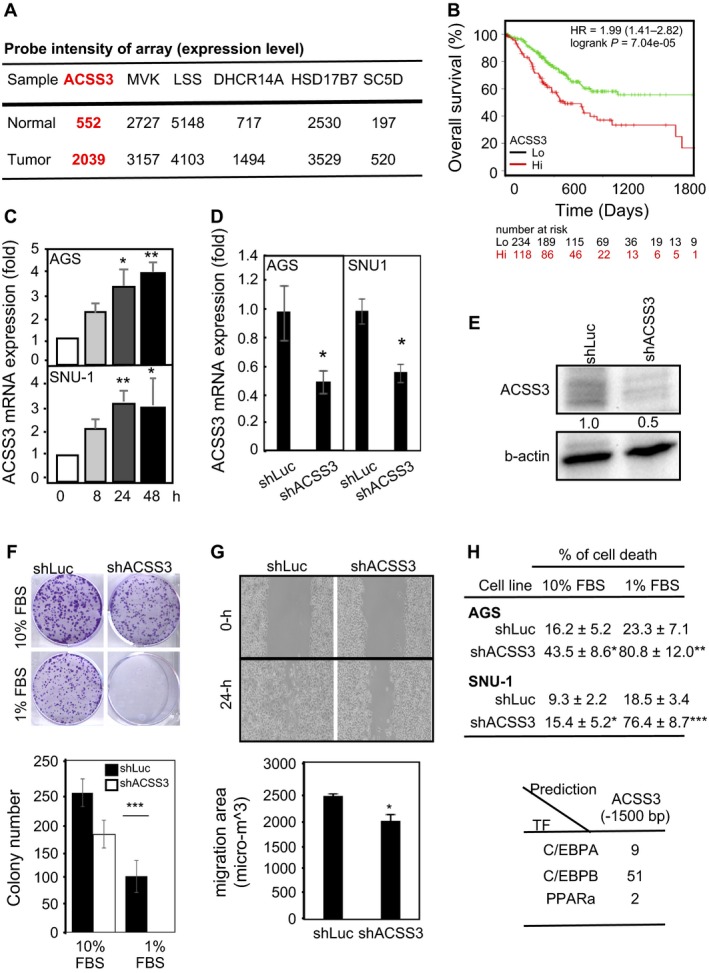
Targeting ACSS3 is a valuable therapeutic strategy. (**A)**. Expression levels (probe intensity of microarray) of five enzymes in cDNA microarray meta‐database from normal parental and tumor tissues. The average probe intensity of the five enzymes shows that ACSS3 in tumor tissue has high levels and increased expression compared to normal parental tissue. (**B)**. Five‐year survival of GCa patients of high (red line) and low (green line) expression of ACSS3. The overall survival analyzed from the TCGA database is consistent with the data analyzed with the KMplotter platform. (**C)**. ACSS3 mRNA is upregulated with the time of starvation in AGS (upper bar‐graph) and SNU‐1 (lower bar‐graph) cells. (**D‐E)**. Knockdown of ACSS3 mRNA (**D**) and protein (**E**) in AGS (left bar‐graph) and SNU‐1 (right bar‐graph) human GCa cell lines. shLuc: short hairpin sequence targeting luciferase, which serves as a control infection. shACSS3: short hairpin sequence targeting ACSS3 gene. (**F)**. Colony‐forming ability of AGS cells with shLuc or shACSS3 knockdown. Upper‐panels are photos of representative experiments, and lower‐panel indicate the quantitation of colony numbers of each experimental group. (**G)**. Migration ability measured by wound‐healing assay on AGS cells with shLuc or shACSS3 knockdown. Upper‐panels are photos of representative experiments, and lower‐panel indicate the quantitation of each experimental group. (**H)**. Cell death measure by PI. Staining following with flowcytometry analysis on AGS and SNU‐1 cells with shLuc or shACSS3 knockdown. 10% FBS indicating regular culture condition, while 1% FBS representing starvation condition. (**I)**. Prediction result of C/EBPs and PPARa binding sequence on ACSS3 5‐promoter (TSS to –1500bps).

The next step is to evaluate the possibility of targeting the genes. The cDNA microarray of KM plotter provides important information about the expression intensities of GCa tumors and their normal parental tissues. As shown in Figure 6A, the average tumor expression signals of ACSS3, DHCR14, and SC5D are higher than the expression in normal parental tissues. However, considering the general impact (DHCR14 has less impact on HER2– patients) and the expression intensity (which is low for SC5D), ACSS3 is considered the most possible target among the five genes.

In order to test this hypothesis, we examined the association between ACSS3 expression and GCa progression using another patient database from The Cancer Genome Atlas (TCGA), which we analyzed using DriverDB.v2. We confirmed that ACSS3 is indeed a GCa prognostic marker (Fig. 6B). We then profiled ACSS3 expression in five human GCa cell lines and found that SNU‐1 and AGS express endogenous ACSS3 (data not shown). Interestingly, we found that the abundance of ACSS3 mRNA increased in low serum culture conditions (1% FBS as starvation conditions). The ACSS3 expression gradually increases to around fourfold in 8‐ to 48‐h of starvation treatment (Fig. 6C). Therefore, we hypothesized that ACSS3 serves as a survival signal during starvation.

We established ACSS3 knockdown (shACSS3) stable transfected cells of AGS and SNU‐1(Fig. 6D, mRNA; Fig. 6E, protein) and then tested cell growth under various conditions to compare with the control (shLuc). We compared the colony forming ability in AGS cells and found that ACSS3 knockdown could suppress colony formation under regular culture (upper‐panel of Fig. 6F; 10% of FBS); however, it's even dramatic under starvation condition (middle‐panel of Fig. 6F; 1% of FBS). While measuring cell mobility ability, we found knockdown ACSS3 inhibited wound‐healing ability under starvation condition (Fig. 6G). Furthermore, knockdown of ACSS3 in AGS and SNU‐1 cells could increase basal level of cell death, and even more dramatic under starvation condition (Fig. 6H).

In order to propose a possible molecular regulation of ACSS3 under starvation conditions, we analyzed the 5‐promoter (TSS ~‐1500 bp) of the ACSS3 genome. We found that it contains 9 C/EBP*α* (CCAAT‐enhancer‐binding proteins alpha), 51 C/EBP*β*, and 2 PPAR*α* binding elements (Fig. 6I). Interestingly, the C/EBP*α* is suggested as a survival signal under starvation conditions 19. This information indicates that the survival effect of ACSS3 under starvation conditions might be due to activation of C/EBP*α*.

In summary, de novo cholesterogenesis is not a major event in GCa progression. However, the intermediates of lower‐mevalonate pathway enzymes play role in GCa prognosis. On the other hand, the ACSS3 expression is a biomarker of GCa prognosis, and targeting ACSS3 might be an effective therapeutic strategy for GCa patients with unmet medical needs.

## Discussion

**Figure 7:**
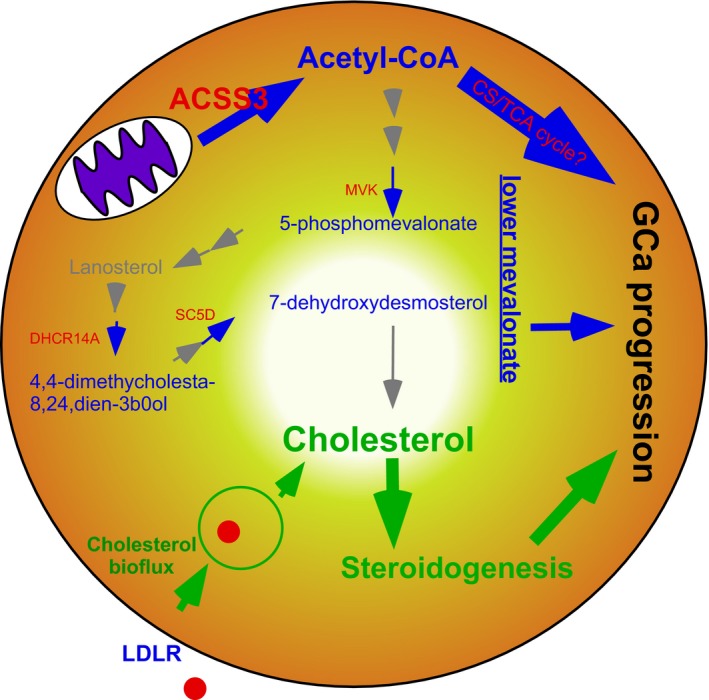
The role of ACSS3 as a prognosis biosignature of GCa. The mitochondrial‐derived acetyl‐CoA (contributed by ACSS3) and intermediates of the lower‐mevalonate pathway enzymes are biosignatures of GCa prognosis (blue). Among them, ACSS3 is a GCa progression confounder. On the other hand, LDLR and related cholesterol bioflux are also key events responsible for GCa progression, by which steroidogenesis occurs 7.

In this study, we determined that ACSS3 is a landmark of cancer progression gatekeeper gene in GCa patients, which is part of the biochemical process for the mitochondrial production of acetyl‐CoA. The cholesterogenesis is initiated by the thiolation of acetyl‐CoA and acetoacetyl‐CoA, which is related to the upper mevalonate pathway. Our results support the notion that the committed‐step enzymes of cholesterogenesis (the upper mevalonate pathway) are not cancer gatekeepers. However, the lipidomes of the lower mevalonate pathway, which are catalyzed by corresponding enzymes, could be biosignatures of GCa progression (Fig. 7).

### The roles of acetyl‐coA in cancer metabolism and progression

The metabolomes are drastically altered during cancer development, which could lead to energy catastrophe and reduce the dependence on oxidative phosphorylation and consumption 20, 21. Acetyl‐CoA has gained attention in cancer research because it participates in epigenetics, posttranslational modification, and cancer cell biomass regulation. Under energy‐deprived conditions where cancer is encountered, hypoxia‐inducible factor 1 (HIF‐1) could block the conversion of pyruvate, inhibit fatty acid *β*‐oxidation, and reduce acetyl‐CoA 22.

Mitochondria are considered as “metabolic checkpoint” organelles that sense the fluctuation of energy in cells 23. The results revealed an adaptation mechanism where mitochondrial ACSS3 can resupply acetyl‐CoA for cancer cells to escape aerobic stress. There is one report that supports this notion. Bjornson et al., predicted that mitochondrial ACSS1 would be an important acetyl‐CoA provider that would be positively associated with hepatocellular carcinoma (HCC) prognosis 24. Another report indicates that the loss of ACSS2 could facilitate GCa progression 25, which is compatible with our results.

In addition to its role as an acetyl‐CoA provider in GCa cells, ACSS3 might also inhibit cancer autophagic activity and escape autophagocide. Some articles reported that the depletion of acetyl‐CoA in cells resulted in increased autophages in yeast 26 and in mammal cells 27. Therefore, there is also a possibility that ACSS3 promotes GCa progression by eliminating autophage stress in the cells.

### Failure of statin trials in GCa patients: Is it the wrong target?

Statin usage (HMGCR blockers and rate‐limiters of the mevalonate pathway) could reduce multiple cancer risks 28. This indicates hope as a possible adjuvant therapy agent for GCa patients. There has been some success of using statins in preclinical GCa models 29, 30, 31, but most clinical trials have failed 32, 33, 34, 35. In this study, we found that HMGCR is not a positive promoter for GCa progression. This indicates that de novo cholesterogenesis is not a major cholesterol provider in GCa. This conclusion also aligns with our previous finding that cholesterol uptake through a lipoprotein/receptor (L/R) route is a source of cholesterol for GCa progression 7.

Although the committed‐step enzymes of cholesterogenesis do not affect GCa progression, the lipidomes produced by lower‐mevalonate enzymes do. We found that MVK, DHCR14, and SC5D affect GCa progression in stage 3, intestinal‐type, and HER2‐negative GCa patients (Figs. 3, 4, 5). To the best of our knowledge, this is the first report that mevalonate‐pathway enzymes are progression promoters. However, some functions of these enzymes have been reported to affect other cellular functions aside from cholesterogenesis. For example, a recent study found that MVK is an innate immune suppressor that mediates toll‐like receptor (TLR)‐induced PI3K activation 36. In addition, Bellezza et al. (2013) found that DHCR14 serves as innate immune modulator by regulating TNF*α* expression 37.

In summary, our study showed that cholesterol supply promotes GCa progression 7, while de novo cholesterol synthesis is irrelevant to GCa progression. These results are consistent with those of Yorket al (2015), who found that cholesterol influx is reduced by activating type I IFN signaling in cancer 38. They also explained the mechanism of cholesterol influx in cancer. Our published work also pointed out increased influx of cholesterol for steroidogenesis, which promotes GCa progression 7. Furthermore, this study indicates that not cholesterogenesis but the lipidomes of the lower mevalonate pathway are promoters of GCa progression.

### The C/EBP*α*‐ACSS3 axis under starvation stress promotes GCa progression

Cancer initiation and progression are often accompanied by hypoxic and nutrient‐deficient stress. In this study, we reported the importance of ACSS3 for GCa progression. ACSS3 has low expression in normal tissue but is significantly upregulated in tumors (Fig. 6A). In this study, we observed ACSS3 upregulation under starvation conditions (Fig. 6C). Furthermore, in vitro study revealed that ACSS3 is critical for cancer cell growth, migration, and death under starvation conditions (Fig. 6F–H). The promoter analyses of the ACSS3 genome showed that there are multiple transcription factor binding sites associated with starvation signals, such as C/EBPs and PPAR*α*. Interestingly, Lu et al. reported that C/EBP*α* is an important signal for overcoming energy starvation stress during hepatocarcinogenesis 19. Therefore, it is very likely that the C/EBP*α*‐ACSS3 axis is an important regulatory mechanism in GCa development or when cancer cells are under energy deprivation stress. Further study of the C/EBP*α*‐ACSS3 axis in tumors would be valuable for understanding most solid tumors under starvation stress.

### Targeting value of ACSS3 in GCa therapy

ACSSs are key enzymes for synthesizing fatty acids through the formation of thioesters with CoA. Three subfamily members are currently recognized in the human genome: ACSS1, ACSS2, and ACSS3. The mitochondrial isoforms ACSS1 and ACSS3 play key roles in the metabolism of acetate for energy production 39. In addition to its ability to induce fatty acid synthesis, ACSS1 is also reported as an epigenetic metabolite that promotes cancer cell survival under hypoxic stress 40. Moreover, it has also been reported that increased ACSS1/2 expression could lead to the import of short‐chain fatty acid (C11), which switches cancer cells from glucose to fatty‐acid utilization 41. This paper suggested that targeting ACSSs might have therapeutic value.

## Conclusion

Considering the intracellular acetyl‐CoA pool, ACSS3 acts as a mitochondrial acetyl‐CoA generator and also serves as a confounder of GCa progression. The de novo cholesterogenesis rate‐limiting step enzymes HMGCS and HMGCR do not promote GCa progression, which explains the failure of clinical trials of statin in GCa patients. This report is the first to indicate ACSS3 as a biomarker of GCa prognosis and that targeting ACSS3 in GCa patients might be therapeutically valuable.

## Availability of data and material

The datasets generated in this study are available at http://kmplot.com/analysis/index.php?p=service&cancer=gastric. All datasets are also available from the corresponding author upon reasonable request.

## Conflicts of Interest

All authors of this manuscript claim no conflicts of interest.

## References

[1] Thrumurthy, S. G. , M. A. Chaudry , I. Chau , and W. Allum . 2015 Does surgery have a role in managing incurable gastric cancer? Nat. Rev. Clin. Oncol. 12:676–682. 26260039 10.1038/nrclinonc.2015.132

[2] Tan, P. , and K. G. Yeoh . 2015 Genetics and molecular pathogenesis of gastric adenocarcinoma. Gastroenterology 149:1153–62 e3. 26073375 10.1053/j.gastro.2015.05.059

[3] Bang, Y. J. , E. Van Cutsem , A. Feyereislova , H. C. Chung , L. Shen , A. Sawaki , et al. 2010 Trastuzumab in combination with chemotherapy versus chemotherapy alone for treatment of HER2‐positive advanced gastric or gastro‐oesophageal junction cancer (ToGA): a phase 3, open‐label, randomised controlled trial. Lancet 376:687–697. 20728210 10.1016/S0140-6736(10)61121-X

[4] Allum, W. H. , J. M. Blazeby , S. M. Griffin , D. Cunningham , J. A. Jankowski , and R. Wong . 2011 Guidelines for the management of oesophageal and gastric cancer. Gut 60:1449–1472. 21705456 10.1136/gut.2010.228254

[5] Foo, M. , and T. Leong . 2014 Adjuvant therapy for gastric cancer: current and future directions. World J. Gastroenterol. 20:13718–13727. 25320509 10.3748/wjg.v20.i38.13718 PMC4194555

[6] Sun, J. , Y. Song , Z. Wang , X. Chen , P. Gao , Y. Xu , et al. 2013 Clinical significance of palliative gastrectomy on the survival of patients with incurable advanced gastric cancer: a systematic review and meta‐analysis. BMC Cancer 13:577. 24304886 10.1186/1471-2407-13-577 PMC4235220

[7] Chang, W. C. , S. F. Huang , Y. M. Lee , H. C. Lai , B. H. Cheng , W. C. Cheng , et al. 2017 Cholesterol import and steroidogenesis are biosignatures for gastric cancer patient survival. Oncotarget 8:692. 27893427 10.18632/oncotarget.13524 PMC5352189

[8] Li, Q. , N. J. Birkbak , B. Gyorffy , Z. Szallasi , and A. C. Eklund . 2011 Jetset: selecting the optimal microarray probe set to represent a gene. BMC Bioinformatics 12:474. 22172014 10.1186/1471-2105-12-474 PMC3266307

[9] Gyorffy, B. , P. Surowiak , J. Budczies , and A. Lanczky . 2013 Online survival analysis software to assess the prognostic value of biomarkers using transcriptomic data in non‐small‐cell lung cancer. PLoS ONE 8:e82241. 24367507 10.1371/journal.pone.0082241 PMC3867325

[10] Chung, I. F. , C. Y. Chen , S. C. Su , C. Y. Li , K. J. Wu , H. W. Wang , et al. 2015 DriverDBv2: a database for human cancer driver gene research. Nucleic Acids Res. 44(D1):D975–D979. 26635391 10.1093/nar/gkv1314 PMC4702919

[11] Cheng, W. C. , I. F. Chung , C. Y. Chen , H. J. Sun , J. J. Fen , W. C. Tang , et al. 2014 DriverDB: an exome sequencing database for cancer driver gene identification. Nucleic Acids Res. 42(Database issue):D1048–D1054. 24214964 10.1093/nar/gkt1025 PMC3965046

[12] Ma, W. L. , C. L. Hsu , M. H. Wu , C. T. Wu , C. C. Wu , J. J. Lai , et al. 2008 Androgen receptor is a new potential therapeutic target for the treatment of hepatocellular carcinoma. Gastroenterology 135:947–955, 55 e1‐5. 18639551 10.1053/j.gastro.2008.05.046 PMC2753209

[13] Ma, W. L. , C. L. Hsu , C. C. Yeh , M. H. Wu , C. K. Huang , L. B. Jeng , et al. 2012 Hepatic androgen receptor suppresses hepatocellular carcinoma metastasis through modulation of cell migration and anoikis. Hepatology 56:176–185. 22318717 10.1002/hep.25644 PMC3673306

[14] Lai, H. C. , C. C. Yeh , L. B. Jeng , S. F. Huang , P. Y. Liao , F. J. Lei , et al. 2016 Androgen receptor mitigates postoperative disease progression of hepatocellular carcinoma by suppressing CD90 + populations and cell migration and by promoting anoikis in circulating tumor cells. Oncotarget 7:46448–46465. 27340775 10.18632/oncotarget.10186 PMC5216809

[15] Ma, W. L. , L. B. Jeng , H. C. Lai , P. Y. Liao , and C. Chang . 2014 Androgen receptor enhances cell adhesion and decreases cell migration via modulating beta1‐integrin‐AKT signaling in hepatocellular carcinoma cells. Cancer Lett. 351:64–71. 24944078 10.1016/j.canlet.2014.05.017

[16] Chen, L. , W. C. Chang , Y. C. Hung , Y. Y. Chang , B. Y. Bao , H. C. Huang , et al. 2014 Androgen receptor increases CD133 expression and progenitor‐like population that associate with cisplatin resistance in endometrial cancer cell line. Reprod. Sci. 21:386–394. 23962788 10.1177/1933719113497281 PMC3936416

[17] Hung, Y. C. , W. C. Chang , L. M. Chen , Y. Y. Chang , L. Y. Wu , W. M. Chung , et al. 2014 Non‐genomic estrogen/estrogen receptor alpha promotes cellular malignancy of immature ovarian teratoma in vitro. J. Cell. Physiol. 229:752–761. 24142535 10.1002/jcp.24495

[18] Hwang, S. H. , H. I. Kim , J. S. Song , M. H. Lee , S. J. Kwon , and M. G. Kim . 2016 The ratio‐based N staging system can more accurately reflect the prognosis of T4 gastric cancer patients with D2 Lymphadenectomy Compared with the 7th American Joint Committee on Cancer/Union for International Cancer Control Staging System. J. Gastric. Cancer. 16:207–214. 28053806 10.5230/jgc.2016.16.4.207 PMC5206310

[19] Lu, G. D. , Y. H. Ang , J. Zhou , J. Tamilarasi , B. Yan , Y. C. Lim , et al. 2015 CCAAT/enhancer binding protein alpha predicts poorer prognosis and prevents energy starvation‐induced cell death in hepatocellular carcinoma. Hepatology 61:965–978. 25363290 10.1002/hep.27593 PMC4365685

[20] Jaworski, D. M. , A. M. Namboodiri , and J. R. Moffett . 2016 Acetate as a metabolic and epigenetic modifier of cancer therapy. J. Cell. Biochem. 117:574–588. 26251955 10.1002/jcb.25305

[21] Edmunds, L. R. , L. Sharma , A. Kang , J. Lu , J. Vockley , S. Basu , et al. 2014 c‐Myc programs fatty acid metabolism and dictates acetyl‐CoA abundance and fate. J. Biol. Chem. 289:25382–25392. 25053415 10.1074/jbc.M114.580662 PMC4155699

[22] Huang, D. , T. Li , X. Li , L. Zhang , L. Sun , X. He , et al. 2014 HIF‐1‐mediated suppression of acyl‐CoA dehydrogenases and fatty acid oxidation is critical for cancer progression. Cell Rep. 8:1930–1942. 25242319 10.1016/j.celrep.2014.08.028

[23] Green, D. R. , L. Galluzzi , and G. Kroemer . 2014 Cell biology. Metabolic control of cell death. Science 345:1250256. 25237106 10.1126/science.1250256 PMC4219413

[24] Bjornson, E. , B. Mukhopadhyay , A. Asplund , N. Pristovsek , R. Cinar , S. Romeo , et al. 2015 Stratification of hepatocellular carcinoma patients based on acetate utilization. Cell Rep. 13:2014–2026. 26655911 10.1016/j.celrep.2015.10.045

[25] Hur, H. , Y. B. Kim , I. H. Ham , and D. Lee . 2015 Loss of ACSS2 expression predicts poor prognosis in patients with gastric cancer. J. Surg. Oncol. 112:585–591. 26381042 10.1002/jso.24043

[26] Eisenberg, T. , S. Schroeder , A. Andryushkova , T. Pendl , V. Kuttner , A. Bhukel , et al. 2014 Nucleocytosolic depletion of the energy metabolite acetyl‐coenzyme a stimulates autophagy and prolongs lifespan. Cell Metab. 19:431–444. 24606900 10.1016/j.cmet.2014.02.010 PMC3988959

[27] Marino, G. , F. Pietrocola , T. Eisenberg , Y. Kong , S. A. Malik , A. Andryushkova , et al. 2014 Regulation of autophagy by cytosolic acetyl‐coenzyme A. Mol. Cell 53:710–725. 24560926 10.1016/j.molcel.2014.01.016

[28] Mullen, P. J. , R. Yu , J. Longo , M. C. Archer , and L. Z. Penn . 2016 The interplay between cell signalling and the mevalonate pathway in cancer. Nat. Rev. Cancer 16:718–731. 27562463 10.1038/nrc.2016.76

[29] Kodach, L. L. , R. J. Jacobs , P. W. Voorneveld , M. E. Wildenberg , H. W. Verspaget , T. van Wezel , et al. 2011 Statins augment the chemosensitivity of colorectal cancer cells inducing epigenetic reprogramming and reducing colorectal cancer cell ‘stemness’ via the bone morphogenetic protein pathway. Gut 60:1544–1553. 21551187 10.1136/gut.2011.237495

[30] Chushi, L. , W. Wei , X. Kangkang , F. Yongzeng , X. Ning , and C. Xiaolei . 2016 HMGCR is up‐regulated in gastric cancer and promotes the growth and migration of the cancer cells. Gene 587:42–47. 27085483 10.1016/j.gene.2016.04.029

[31] Cheng‐Qian, Y. , W. Xin‐Jing , X. Wei , G. Zhuang‐Lei , Z. Hong‐Peng , X. Songde , et al. 2014 Lovastatin inhibited the growth of gastric cancer cells. Hepatogastroenterology 61:1–4. 24895782

[32] Konings, I. R. , A. van der Gaast , L. J. van der Wijk , F. E. de Jongh , F. A. Eskens , and S. Sleijfer . 2010 The addition of pravastatin to chemotherapy in advanced gastric carcinoma: a randomised phase II trial. Eur. J. Cancer 46:3200–3204. 20727735 10.1016/j.ejca.2010.07.036

[33] Kim, S. T. , J. H. Kang , J. Lee , S. H. Park , J. O. Park , Y. S. Park , et al. 2014 Simvastatin plus capecitabine‐cisplatin versus placebo plus capecitabine‐cisplatin in patients with previously untreated advanced gastric cancer: a double‐blind randomised phase 3 study. Eur. J. Cancer 50:2822–2830. 25218337 10.1016/j.ejca.2014.08.005

[34] Browning, D. R. , and R. M. Martin . 2007 Statins and risk of cancer: a systematic review and metaanalysis. Int. J. Cancer 120:833–843. 17131313 10.1002/ijc.22366

[35] Bujanda, L. , A. Rodriguez‐Gonzalez , C. Sarasqueta , E. Eizaguirre , E. Hijona , J. J. Marin , et al. 2016 Effect of pravastatin on the survival of patients with advanced gastric cancer. Oncotarget 7:4379–4384. 26735890 10.18632/oncotarget.6777 PMC4826212

[36] Akula, M. K. , M. Shi , Z. Jiang , C. E. Foster , D. Miao , A. S. Li , et al. 2016 Control of the innate immune response by the mevalonate pathway. Nat. Immunol. 17:922–929. 27270400 10.1038/ni.3487 PMC4955724

[37] Bellezza, I. , R. Roberti , L. Gatticchi , R. Del Sordo , M. G. Rambotti , M. C. Marchetti , et al. 2013 A novel role for Tm7sf2 gene in regulating TNFalpha expression. PLoS ONE 8:e68017. 23935851 10.1371/journal.pone.0068017 PMC3720723

[38] York, A. G. , K. J. Williams , J. P. Argus , Q. D. Zhou , G. Brar , L. Vergnes , et al. 2015 Limiting cholesterol biosynthetic flux spontaneously engages type I IFN signaling. Cell 163:1716–1729. 26686653 10.1016/j.cell.2015.11.045 PMC4783382

[39] Watkins, P. A. , D. Maiguel , Z. Jia , and J. Pevsner . 2007 Evidence for 26 distinct acyl‐coenzyme a synthetase genes in the human genome. J. Lipid Res. 48:2736–2750. 17762044 10.1194/jlr.M700378-JLR200

[40] Gao, X. , S. H. Lin , F. Ren , J. T. Li , J. J. Chen , C. B. Yao , et al. 2016 Acetate functions as an epigenetic metabolite to promote lipid synthesis under hypoxia. Nat. Commun. 7:11960. 27357947 10.1038/ncomms11960 PMC4931325

[41] Yun, M. , S. H. Bang , J. W. Kim , J. Y. Park , K. S. Kim , and J. D. Lee . 2009 The importance of acetyl coenzyme A synthetase for 11C‐acetate uptake and cell survival in hepatocellular carcinoma. J. Nucl. Med. 50:1222–1228. 19617323 10.2967/jnumed.109.062703

